# An Energy Efficient Thermally Regulated Optical Spectroscopy Cell for Lab-on-Chip Devices: Applied to Nitrate Detection

**DOI:** 10.3390/mi12080861

**Published:** 2021-07-22

**Authors:** Benjamin J. Murphy, Edward A. Luy, Katerina L. Panzica, Gregory Johnson, Vincent J. Sieben

**Affiliations:** 1 Department of Electrical and Computer Engineering, Dalhousie University, 1360 Barrington Street, Halifax, NS B3H 4R2, Canada; bn744579@dal.ca (B.J.M.); eddy.luy@dal.ca (E.A.L.); panzicak@mcmaster.ca (K.L.P.); 2 RBR Limited, 359 Terry Fox Drive, Ottawa, ON K2K 2E7, Canada; greg.johnson@rbr-global.com

**Keywords:** microfluidic, lab on chip, nitrate, absorbance, heaters, colorimetric

## Abstract

Reagent-based colorimetric analyzers often heat the fluid under analysis for improved reaction kinetics, whilst also aiming to minimize energy use per measurement. Here, a novel method of conserving heat energy on such microfluidic systems is presented. Our design reduces heat transfer to the environment by surrounding the heated optical cell on four sides with integral air pockets, thereby realizing an insulated and suspended bridge structure. Our design was simulated in COMSOL Multiphysics and verified in a polymethyl methacrylate (PMMA) device. We evaluate the effectiveness of the insulated design by comparing it to a non-insulated cell. For temperatures up to 55 °C, the average power consumption was reduced by 49.3% in the simulation and 40.2% in the experiment. The designs were then characterized with the vanadium and Griess reagent assay for nitrate at 35 °C. Nitrate concentrations from 0.25 µM to 50 µM were tested and yielded the expected linear relationship with a limit of detection of 20 nM. We show a reduction in energy consumption from 195 J to 119 J per 10 min measurement using only 4 µL of fluid. Efficient heating on-chip will have broad applicability to numerous colorimetric assays.

## 1. Introduction

Several environmental sensors are reagent-based and rely on colorimetric approaches to achieve ultra-low limits of detection; for example, nanomolar levels of ammonium, nitrate, nitrite, phosphate, iron, silicate, manganese, and sulfide as reviewed recently [1]. However, reagent-based systems, such as colorimetric sensors, are sensitive to the temperature under which the reaction kinetics occur. For example, Schnetger et al. studied the reaction kinetics of the nitrate vanadium reduction approach and if heated, the reaction can be sped up from hours to minutes [2]. As one of the limiting nutrients, nitrate monitoring is vital to understanding the health and dynamics of aquatic ecosystems. Concentrations of nitrate in the ocean can vary from 0.06 to 31 mg L^−1^ (1–500 µM) [3]. Anthropogenic sources of nitrate, such as agriculture fertilizer runoff into waterways, can lead to eutrophication, shifting ecosystems, and undesirable blooms [4,5]. Further, the World Health Organization reports that concentrations of nitrate in excess of 50 mg L^−1^ (806 µM) are dangerous for human consumption [6]. Therefore, routine monitoring of nitrate concentrations in waterways is important for providing early warning of elevated nitrate levels. Traditionally, such nitrate measurements are taken by manually gathering samples and testing them in laboratories that support benchtop auto-analyzers; for example, the AA500 by SEAL Analytical Inc. Preserving and transporting the samples to the laboratory hinders widespread and routine monitoring of nitrate levels, as it is labor- and resource-intensive. For this reason, several in situ sensors have been developed to improve the spatial and temporal resolution of nitrate measurements [7,8,9,10].

Various techniques are used for the detection of nitrate, including: optical spectroscopy in the ultraviolet (UV) region [9,11], ion-selective electrodes (ISE) [12], liquid chromatography [13], and colorimetric reagent-based assays [7]. Nitrate inherently absorbs strongly in the UV spectrum at 220 nm, and therefore, optical absorbance can be used to measure nitrate concentrations directly. Commercial UV-nitrate sensors, such as the Seabird SUNA or the YSI EXO NitraLED, are readily available. These sensors are appealing in their simplicity, but their measurements are also influenced by matrix effects such as water color and other absorbing species in the UV spectrum, as well as water turbidity [14]. In such systems, the sensor must delineate the attenuation and absorbance coefficients, which can be non-trivial with uncharacterized and potentially widely varying samples [15]. Nitrate ion-selective electrodes have response times as low as 2 s [16] and can detect concentration ranges as wide as 4.96–6.2 × 10^5^ µg L^−1^ (0.08–1 × 10^4^ µM). However, electrochemical nitrate measurements tend to drift significantly over time and have short lifespans. Recent efforts by Hassan et al. have seen the drift reduced to 16.6 µVs^−1^ with a sensitivity of −55.1 mV/decade, but this sensor has a limited lifespan of 8 weeks, making it impractical for long-term in situ monitoring [17]. The drift of the measured voltages would increase the nitrate reading to ten times its original value after 55 min of continuous operation. Commercial nitrate ISE systems, such as YSI’s EXO Nitrate, have a sensitivity of 52 to 62 mV/decade and require frequent and daily calibration. Alternatively, chromatography setups are not easily ported outside of a laboratory setting, as the columns may require changing between use or reconditioning processes that increase the number and volumes of chemicals taken to the environment [18]. For example, reducing nitrate to nitrite using a cadmium column and converting the nitrite to Azo dye with the colorimetric Griess assay is the gold standard method [10,11]. The cadmium column used for nitrate reduction in Yaqoob et al. required 10 mL of fluid to flush the column between each use [19]. Therefore, acquiring 1000s of measurements would require 10s of liters to be deployed for supporting the column reduction step, not counting the copper reconditioning required. Liquid flow injection analysis (FIA) schemes permit tens of samples per hour to be measured [20,21], whereas stopped flow schemes permit a few samples per hour [10,22]. In both cases, to minimize reagent consumption, lab-on-chip technology and small microchannels have been utilized to perform similar assays. Pioneering work by Petsul et al. in 1999 demonstrated an FIA system driven by electro-osmotic flow (EOF) for nitrate using microfluidic technology on the benchtop, using only 2 µL per measurement [23]. A decade later, Beaton et al. deployed a stop-flow microfluidic and in situ nitrate sensor using the cadmium column reduction and Griess assay approach, reducing the column holdup volume to 80 µL [10]. While this method is both accurate and reliable, the cadmium column is toxic and presents challenges in handling and disposal.

Recent efforts have seen the solid phase cadmium column replaced with a reagent reduction based on the less toxic vanadium (III) chloride solution [2]. The vanadium reduction reaction is considerably slower than the cadmium reduction, taking multiple hours at room temperature. Schnetger et al. studied the reaction kinetics of the vanadium reduction approach and if heated from 22 °C to 45 °C, the reaction can be sped up from 390 min to 40 min for achieving over 95% color development [2]. As with most reagent-based assays, the reaction kinetics of the vanadium–Griess method occur faster by heating, although if the goal is to make an in situ instrument, this comes with an energy cost. 

Performing reagent assays quickly under in situ conditions, such as the vanadium nitrate reduction, will likely require on-chip heating. Several methods for heating small fluid volumes on lab-on-chip devices have been studied [24], such as microwave heating, heating by chemical reaction, and joule heating [25]. Microwave heating provides fast temperature responses with heating times in the 10s of milliseconds [26]; however, safely waveguiding the energy and establishing uniform spatial distributions of the electromagnetic fields (EMF) in the reactor cavity is challenging [27]. Chemical reactions can provide temperature control using exothermic reactions; for instance, flowing a 11:1 volumetric mixture of water to sulfuric acid in a parallel channel can generate enough heat to bring a 19 µm by 54 µm channel to 36 °C [24]. However, such approaches require additional reagents to be deployed with the sensor, increasing the size of the system and reducing energy efficiency through necessitating additional pumping [25]. Alternatively, on-chip resistive heaters are relatively low-cost, require less support equipment, and can be realized in customizable shapes, thereby making Joule heating the most common method. These microfabricated heaters are available in a wide array of materials including platinum, doped silicon, and titanium [28]. Using a platinum joule heater, Hoang et al. were able achieve ramp rates of 10 °C per second to perform rapid polymerase chain reaction, cycling temperatures from 60 to 94 °C in a 0.6 µL chamber using only 1 W of power [29]. More simply, isothermal control has been used to accelerate reagent-based assays with resistive heaters. In the case of the nitrate vanadium reduction method, Nightingale et al. performed in situ nitrate measurements with an off-the-shelf polyimide thin-film heater, where the sensor consumed 1.5 W of power with 0.5 W used for heating [7]. While improving reaction kinetics provides clear gains for reagent-based sensors, few studies have focused on the energy efficiency of delivering thermal energy to the microchannel spectroscopy cell. 

Here, we improve the energy efficiency of thermally regulated optical cells by incorporating on-chip insulation. Prior work by Zhao et al. [30] and Dijkstra et al. [31] showed that a suspended microfluidic channel surrounded by an air cavity can be used to efficiently couple thermal energy into a fluid. For example, the system presented by Zhao et al. was able to heat a suspended microfluidic channel to 119.4 °C using only 206.9 mW [30]. Such suspended structures have been utilized as flow sensors by either measuring temperature deviation in the insulated channel [31] or vibrations caused by µ-Coriolis effects [32]. To the best of the authors’ knowledge, the use of free-hanging channels has not been applied to performing on-chip optical spectroscopy, where light is coupled into and out of a long suspended and heated microchannel. 

**Figure 1:**
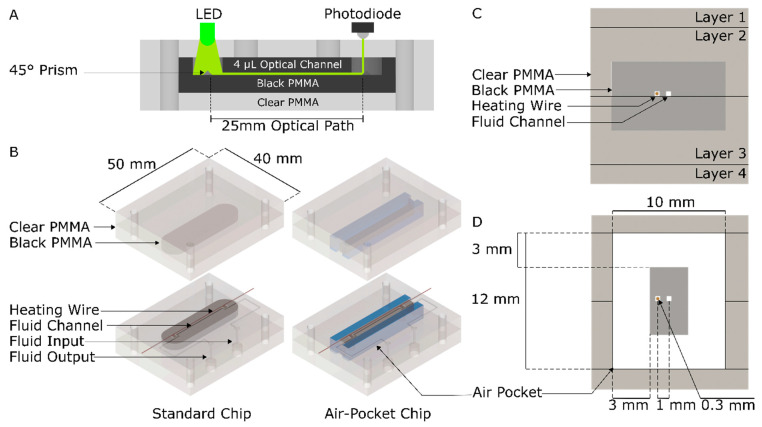
(**A**) Cross-section of the inlaid absorbance cell, identifying the light-emitting diode (LED) light source, the reflecting prisms, the optical path, and the hybrid black and clear polymethyl methacrylate (PMMA) chip design. (**B**) The 3D rendering of the chip with the top and bottom halves of the chip separated to show its internal features for both the standard and insulating air pocket designs. The air pockets are highlighted in blue. A nichrome wire inserted beside the channel provides thermal regulation of the absorbance cell. (**C**) Cross-section of the standard chip design, showing the layout of the wire and channel in the inlay. (**D**) Cross-section of the insulated chip design, showing air pockets added around the optical cell to create an integral cavity and increase thermal resistance to the environment.

We propose and validate a novel “suspended bridge” optical cell that reduces energy consumption from 195 Joules (non-insulated) to 119 Joules (insulated) per 10 min nitrate measurement. Figure 1 shows our microfluidic optical absorbance cell with integrated heaters in both the standard and insulated configurations. We base the design on our “inlaid” optical cell approach, as shown in Figure 1A, where black polymethyl methacrylate (PMMA) is embedded within clear PMMA to provide the necessary optical isolation for absorbance measurements [22]. The inlaid absorbance cell in Figure 1A depicts the light guiding through the microchannel optical cell to make colorimetric measurements. The optical channel, based on our previous work [22], is surrounded in dark PMMA to prevent extraneous light from entering the channel and interfering with measurements. The light from the light-emitting diode (LED) shines down through a clear hole in the dark PMMA, reflects off of a 45° prism, passes through the 25 mm optical channel, reflects off of a second 45° prism, and finally, reaches the photodiode. Figure 1B compares the standard and insulated on-chip resistive heater designs using a 3D rendering, with the top and bottom halves of the chip separated to highlight the internal details of the chip. The heater, fluid channel, fluid input, and fluid output are labeled on the standard chip. Figure 1C,D provide cross-sectional views of the standard and insulated designs. Figure 1C illustrates the standard configuration of the black PMMA inlayed in clear PMMA and fluid channel, as well as the added heater wire. Figure 1D shows the integral air pockets around the optical absorbance cell, forming an insulated bridge design. The approach conserves energy by increasing the thermal resistance to the local environment. Placing the air pockets close to the sensing channel permits rapid thermal control and minimizes thermal mass. The inlaid absorbance cell augmented with integral insulated heating allows for low volume, low power, thermal regulation, and on-chip colorimetric spectroscopy. We demonstrate absorbance measurements on 4 µL of fluid with optical path lengths of 25 mm, thermally controlled between temperatures from ambient or room temperature, up to 55 °C. While we characterized our design and demonstrated utility for nitrate measurements, this approach will be broadly applicable to colorimetric analyzers that perform reactions on-chip that could benefit from efficient thermal regulation.

## 2. Materials and Methods

### 2.1. Chip Fabrication

Our design of a thermally isolated microfluidic absorbance cell was made from four substrates of 6 mm-thick PMMA (0A000, Acrylite, Sanford, ME, USA) using a modified approach to what was described by Luy et al. [22]. Modifications to the published procedure were made to incorporate a heating element embedded between the layers and to make integral air pockets. First, an LPKF S103 micro-mill (LPKF, Garbsen, Germany) was used to mill recesses into the middle two clear PMMA substrates to allow black PMMA (9M001, Acrylite, Sanford, ME, USA) to be inlaid into them. The black PMMA was cut into the shape of the recessed cavities in the clear PMMA using a 50-watt Epilog Mini laser cutter (Epilog, Golden, CO, USA). Both the clear and black PMMA were exposed to chloroform to soften the plastic and then, the black PMMA was pressed into the clear PMMA base using an LPKF Multipress II for 3 h at 116 °C and 43 N cm^−2^. The inlaid optical cell ensured that the final absorbance measurements were conducted on the fluid alone, preventing extraneous or scattered light from entering the optical channel.

The inlaid substrate (black and clear PMMA interwoven) was then leveled by shaving 200 µm off from the top of the sheet using an LPKF micro-mill. This was carried out to ensure a smooth surface with substrate thickness uniformity and a channel depth accuracy of less than 10 microns. Microchannel features were cut into the leveled inlaid PMMA with the LPKF S103 micro-mill; nominal fluid channel dimensions were 400 µm deep and 400 µm wide. The individual chip plates were then cut out with the Epilog Mini Laser. Both interior plates (labelled layers 2 and 3 in Figure 1C) were then sanded and scrubbed to ensure the surfaces to be pressed together were both clean and deburred. Next, these middle two substrates were exposed to chloroform vapor for 45 s to soften them for bonding. A 300 µm diameter nichrome-80 wire was placed into the designated pre-cut channel, then the two substrates were aligned with dowels and hand-pressed together. The combined substrates were placed in the automated LPKF Multipress II for 135 min at 85 °C and 625 N cm^−2^ to complete the bond. After the middle two plates were pressed, air pockets were milled into the chip around the optical absorbance channel to create a bridge containing the optical channel and heater wire, as shown in Figure 1B,D. Finally, features were milled into the top and bottom plates using the LPKF S103 micro-mill and then were cut out using the Epilog Mini Laser. The layer 1 and 4 plates were screwed onto the top and bottom of the chip, thereby enclosing the air around the bridge and creating an air pocket. To create the standard chip, the procedure was the same, except air pockets were not created around the optical channel and heating wire.

The initial design was constructed from PMMA, which limits the range of temperatures possible, a constraint imposed by its glass transition temperature, *T*_g_ of 100–130 °C [33]. This is acceptable for nitrate detection, which does not require temperatures higher than 50 °C. Other materials with higher glass transition temperatures such as cyclic olefin copolymers (*T*_g_ = 177 °C [34]) could be used to apply a similar design to other purpose, e.g., polymerase chain reaction, that may require higher temperature ranges. Looking forward, advances in 3D printing technology at the micron level could further improve the speed of the rapid prototyping process [35], including with multi-material stacks [36]. In this case, the 3D printed microfluidic chips would eliminate the need to mill separate layers then align and press them. This would remove some of the more time consuming and failure prone aspects of chip construction, while simultaneously printing the integral air pockets in chips that are constructed from more robust materials such as ceramics.

COMSOL Multiphysics 5.5 (COMSOL, Stockholm, Sweden) simulations were based on a nichrome heater wire with a resistivity of 1.08 µΩ·m, a diameter of 300 µm, and a length of 5 cm that culminate in a wire resistance of 0.76 Ω. The experimentally measured resistances were in agreement at 0.78 Ω for the standard chip and 0.77 Ω for the insulated chip.

### 2.2. Reagent Preparation 

To prepare the modified Griess reagent, 2.5 g of vanadium (III) chloride, 1.25 g of sulfanilamide, 15 mL of concentrated HCl, and 0.125 g of NEDD (N-(1-aphthyl)ethylenediamine dihydrochloride) were dissolved in Milli-Q water to reach a final volume of 500 mL. Chemicals and reagents used to make modified Griess reagent were sourced from Fisher Chemical (Waltham, MA, USA). The reagent was stored in a dark refrigerator at 4 °C to keep the reagent from degrading between uses [37]. When being used, the reagent bottle was wrapped in tinfoil to maintain the dark environment. Nightingale et al. found that the modified Griess reagent remains functional for at least 9 months stored at room temperature [7]. 

Nitrate standards were prepared from a 1000 µM stock made by diluting 14 mL of 100 ppm nitrogen as 442.68 ppm nitrate stock (NO_3_-N, R5457, RICCA Chemical Company, Arlington, TX, USA) to 100 mL using Milli-Q water. The 1000 µM stock was then sequentially diluted to create the standards used in chip calibration. Eight concentrations of nitrate were applied to the thermally regulated optical cell for characterization. The concentrations used were 0.25 µM, 0.5 µM, 1 µM, 2 µM, 5 µM, 10 µM, 25 µM, and 50 µM, which were tested in ascending order, with each sample tested in triplicate to ensure repeatability. 

### 2.3. Characterization Procedure

Two sets of standard and insulated chips were created—one set to measure temperature and one set to validate chemistry. The temperature measurement chips were identical to the chemistry chips, except for an extra thermocouple port in the center of the chip for measuring channel temperature. The temperature chips were used to determine electrical current setpoints, as well as the time it would take to achieve the steady-state temperatures. Temperature measurements were also acquired with a FLIR ONE Pro (FLIR, Wilsonville, OR, USA) infrared camera with an accuracy of ±2 °C [38].

Absorbance measurements were performed by automated fluid handling using two syringe pumps (Cavro XC, P/N 20 740 556-C, Tecan Systems, Männedorf, Switzerland). Absorbance measurements were performed using a green LED as the light source, centered at 527 nm with a FWHM of approximately 50 nm (C503B, Cree LED, Durham, NC, USA). The detector was a photodiode with a built-in 320 MΩ transimpedance gain amplifier (TSL257, AMS-TAOS Inc., Plano, TX, USA) that was used to acquire the light transmitted through the fluid path. One pump injected the sample and the other injected the reagent. The two syringe pumps delivered 1.5 mL of Griess reagent and 1.5 mL of sample or milli-Q water, each at a flow rate of 1.5 mL/min, into a mixing chip to create a blank sample. The mixing chip consisted of two input ports that merged into a 43 cm long serpentine channel with width and depth of 400 µm. The sample and reagent mixed while traveling through the channel then exited the mixing chip and entered the optical cell through a 58 cm long and 762 µm diameter fluoropolymer (FEP) tubing (IDEX 1520XL, Northbrook, IL, USA). The transit time was 7 s for the fluid to reach the optical cell after it began the mixing process, 69 µL in the chip and 265 µL in the tube. Multiple samples were introduced to the chip in an automated fashion using a selector valve (Vici Cheminert C65Z 10-port selector valve, Model No. C65-3710IA, Valco Instruments Co. Inc. Houston, TX, USA). The automated switching between nitrate concentrations and Milli-Q blanks was controlled with a custom script language developed in C. Electrical current was passed through a 300 µm diameter nichrome 80 wire using a CS1305 benchtop power source (Circuit Specialists, Tempe, AZ, USA) to heat the optical cell. A stopped flow approach was used, and the fluid was kept at the temperature setpoint for 10 min to allow the nitrate-to-nitrite reduction, as well as the Griess reaction to occur and the Azo dye to form. Absorption measurements were continuously acquired from the fluid in the optical channel to determine the dye’s concentration over time. The fluid was then pumped out of the chip into the waste container. The optical channel was then flushed with 15 mL of a 1 part milli-Q and 1 part Griess reagent mixture to clear the remaining sample and reduce crosstalk between samples.

### 2.4. Data Analysis

Absorbance of nitrate concentration was calculated using the Beer–Lambert law. The process was as follows. First, the LED was turned off, and the voltage output of the photodiode at the end of the absorbance cell was recorded over 15 s and averaged to obtain *V_Dark_*. Second, the blank was introduced to the measurement channel by delivering 1 part milli-Q water and 1 part modified Griess reagent mixture, which was allowed to develop for 10 min. The voltage output over the last 15 s of the development time was averaged to obtain *V_Blank_*. Third, the sample was introduced by injecting a mixture of 1 part known concentration of nitrate and 1 part modified Griess reagent to obtain *V_Sample_*. *V_Dark_* was subtracted from both *V_Sample_* and *V_Blank_* to negate the effect of light reaching the photodiode from sources not passing through the fluid channel.

(1) $${V^{\prime }}_{Sample}=V_{Sample}-V_{Dark}\;\;\;||\;\;\;{V^{\prime }}_{Blank}=V_{Blank}-V_{Dark}$$

The modified sample and blank voltages are denoted by *V’_Sample_* and *V′_Blank_*. Finally, the negative logarithm is taken of the modified sample voltage divided by the modified blank voltage to obtain the absorbance value of the known concentration of nitrate. The Beer–Lambert law holds that the absorbance of a medium (*A*) is equal to the concentration (*c*) of the absorbing species in that medium multiplied by the optical path length (*l*) multiplied by the molar attenuation coefficient of the species (ε). The molar attenuation coefficient is also a function of the wavelength (*λ*) of the light passing through the absorbing medium and, by extension, so is the absorbance. From this equation, we can expect a linear relationship between absorbance and nitrate concentration at our fixed peak wavelength of 527 nm.

(2) $$A\left(\lambda \right)=\varepsilon \left(\lambda \right)cl=-log_{10}\left(\frac{{V^{\prime }}_{Sample}}{{V^{\prime }}_{Blank\;}}\right)$$

The optical cell was studied in our previous work using a similar 25 mm path length inlaid absorbance cell [22]. The cell was simulated in OpticStudio 20.3.2 (Zemax, Kirkland, WA, USA) to determine the optical losses of the system, where the light enters the chip, passes through a blank sample in the fluid channel, and then leaves the chip. The LED light source reports a range of total light intensity from 16.8 cd to 90.5 cd. Using an average LED light intensity of 53.7 cd, the peak intensity was found to be 6.7 lumens/cm^2^ (11 mW/cm^2^) as the light entered the chip and 0.57 lumens/cm^2^ (0.97 mW/cm^2^) as the light exited the chip. For these calculations, a luminous efficiency of 588 lumens/W was used at the LED output peak wavelength of 527 nm. Zemax raytracing calculations, thus, indicate the optical losses of a 25.4 mm inlaid optical cell are more than 90% of the incident light.

The photodiode detector has a final irradiance responsivity of 1.56 V/(µW/cm^2^) arising from the on-board transimpedance gain of 320 MΩ at the LED peak wavelength as reported in its datasheet. When the photodiode reports 4.0–4.5 V, below saturation (e.g., on a blank), this equates to an optical intensity of 2.6–2.9 µW/cm^2^. Even with 90% optical loss through the inlaid cell, the LED provides 3 orders of magnitude more light intensity at the detector side (~1 mW/cm^2^) than required. Empirically, we observe that misalignment of the LED and photodiode to the chip, as well as fabrication imperfections, lead to as much as 2 orders of magnitude of optical loss. The LED is typically driven at 1–10% of its maximum intensity using a constant current source, depending on alignment, which was sufficient for absorbance spectroscopy measurements.

## 3. Results and Discussion

The heater design was simulated in COMSOL, implemented, and then, evaluated to determine thermal performance. Following characterization of the microfluidic chip design, the suspended absorbance cell was used to measure nitrate concentrations and validate nanomolar performance with suitable reaction times.

### 3.1. Heater Simulation

**Figure 2:**
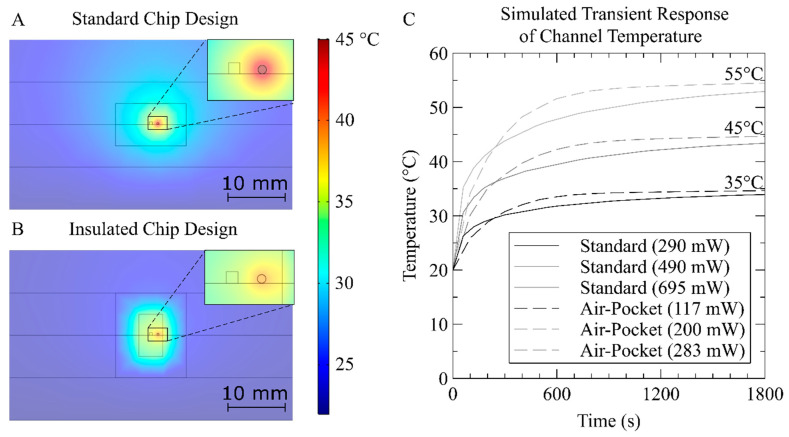
(**A**) Simulated cross-sectional heat distribution in the chip at steady-state conditions without insulating air pockets. The temperature setpoint is 35 °C at the center of the microchannel and required 0.618 A supplied to the heater, which itself attained a temperature of 41.7 °C. (**B**) Simulated cross-sectional heat distribution in the chip at steady-state conditions with insulating air pockets. The same 35 °C channel setpoint required 0.393 A supplied to the heater, with the heater reaching 37.8 °C. (**C**) Transient response of the simulated chip without air pockets (solid lines) and with air pockets (dashed lines) for the first 30 min, showing improved responsivity and energy efficiency of the insulated design. Simulations details described in text.

Figure 2 compares the simulated thermal response of the standard and insulated optical cell designs with a temperature setpoint of 35 °C in the fluid channel. The cross sections of the center of both chips are shown in Figure 2A,B to illustrate the heat distribution under steady state conditions. The scale bar, shown to the right, covers the temperature range from the boundary conditions of room temperature (Blue −20 °C) to the maximum in this instance near the heater (Red −45 °C). The inset shown in the top right corner of Figure 2A,B represents a magnified view of the center of each chip. In the insets, the square geometry is the channel created from micro-milling and the circular geometry is the heater wire. As expected, the highest temperature in both chips is the heater and the temperatures decrease in both chips toward the edges or boundary conditions. When the center of the microfluidic channel is set to 35 °C, the nichrome wire heater must be set to 37.8 °C in the insulated chip, whereas the standard chip wire must be set to 41.7 °C to account for the lack of insulation.

Driving the heater to a higher temperature requires more current or power, and in the simulation, the air pocket design reduces the power required to maintain the channel temperature at 35 °C from 290 mW to 117 mW. Similarly, with a setpoint of 45 °C, the power is reduced from 490 mW to 200 mW, and at 55 °C, from 695 mW to 283 mW. The reductions in power required from the standard to the insulated chip at 35 °C, 45 °C, and 55 °C were 59.6, 59.2, and 59.3%, respectively. This represents a significant reduction in power usage for the range of targeted temperature setpoints suitable for reagent-based assays. While Figure 2 was simulated at 20 °C ambient in air, this heater is intended for in situ deployments in waters that can be as low as 4 °C ambient. Figure S1 in Section A of the supplementary material shows the simulated results for the identical chip designs immersed in water at 4 °C. In this case, our insulated design shows a greater efficiency improvement, and we see the power required to maintain 35 °C, 45 °C, and 55 °C drops from 727 mW, 965 mW, and 1200 mW (non-insulated) to 255 mW, 340 mW, and 428 mW (insulated), or 65.0, 64.8, and 64.5% saving. The above power values required for our heater design are in line with Martinez-Quijada et al., who showed clean-room fabricated thin-film heaters that consumed 1130 mW to elevate a 1 cm chip to 95 °C in 22 °C ambient air [39]. When we drive our insulated heater design to 95 °C, the simulated power consumption is comparable at 900 mW. Therefore, our design shows comparable performance to cleanroom-fabricated devices, but is implemented with more readily available rapid prototyping approaches.

The heat distribution across the fluid channel cross-section in the insulated chip is also more uniform than in the standard chip. In the case of a fluid channel setpoint of 35 °C, where the entire chip is immersed in air at 20 °C, we see a change in temperature of 1.06 °C from one side of the channel to the other in the standard chip, and 0.55 °C in the insulated chip. Repeating the simulation for a chip immersed in water at 4 °C, we note a greater change from one end of the channel to the other: specifically, 2.63 °C in the standard chip and 1.17 °C in the insulated chip. The thermal gradient across the fluid channel cross-section arises from the thermal resistances to the boundary conditions; please see Figure S3 in the supplementary material Section C on the electrical equivalence thermal modelling of our system as well as Table S1 for the material properties used to calculate the thermal impedances. In the standard chip, the temperature drops off gradually over the full distance between the heater and the chip boundary/edge, whereas in the insulated chip, the elevated temperature is confined to the optical cell with a steep gradient across the air pockets. A more uniform temperature profile in the insulated design leads to more consistent reaction kinetics with reagent-based analyzers.

Figure 2C compares the simulated transient response of both chips. The simulated chips began at 20 °C and were heated to microchannel temperature setpoints of 35 °C, 45 °C, and 55 °C using constant currents of 0.618 A, 0.803 A, and 0.956 A for the standard chip and 0.393 A, 0.513 A, and 0.610 A for the insulated chip, respectively. For the first 250 s, the center of the fluid channel has a higher temperature in the standard chip than in the insulated chip. This is an expected result, as the heater in the insulated chip is driven at a lower input power, taking longer to heat the immediate thermal mass of the PMMA plastic. This causes the temperature of the fluid to rise slower during the initial heating phase. However, the air cavity in the insulated design introduces a significant thermal resistance, improving the thermal energy retention, allowing the temperature to overtake the standard design at approximately 5 min. The standard chip reached 95% of the temperature setpoint in 33 min, while the insulated chip required only 17 min to plateau. The response is similar using transient analysis of an equivalent RC circuit, as shown in Figure S4 in section C of the supplementary material.

The faster transient response of the insulated design can further improve its energy efficiency, as less time is required to attain steady-state temperature. To achieve steady state, the standard chip required 33 min and the insulated chip required 17 min, with both chips then holding for an additional 10 min of reaction time. Typical reagent-based colorimetric analyzers that perform stop flow schemes allow color development to occur over at least 2 min, often waiting much longer [10,37]. Faster temperature response is particularly advantageous when an in situ sensor is acquiring measurements infrequently, where the sensor will be put into sleep mode most of the time. When considering the heating up time plus the hold time, the standard chip requires 0.75 kJ, 1.26 kJ, or 1.79 kJ for infrequent measurements to raise the temperature from 20 °C to 35 °C, 45°C, or 55°C in air, respectively. Similarly, the insulated chip requires 0.19 kJ, 0.32 kJ, or 0.46 kJ for the same temperatures, respectively. For each of the temperature setpoints, this is a 74.6, 74.4, 74.4% reduction in the energy required, respectively. When heating from 4 °C to 35 °C, 45°C, or 55 °C in water, the gains are more substantial. The energy reduction from the standard chip to the insulated chip is 1.88 kJ down to 0.41 kJ (4 °C to 35 °C), from 2.49 kJ down to 0.55 kJ (4 °C to 45 °C), and from 3.11 kJ down to 0.69 kJ (4 to 55 °C). For each of the temperature setpoints, this is a 78.0, 77.9, 77.7% reduction in the energy required, respectively. We note that the above is a unique case in that open-loop direct current control of the heater is implemented; however, the system can be driven in an underdamped mode, provided the glass transition temperature of the plastic material is not exceeded.

Future work will see a closed-loop implementation of our design and associated analysis. The addition of a proportional–integral–derivative (PID) temperature controller would allow the channel to be brought to and held at a setpoint temperature faster. This would be vital to monitoring nitrate levels in waterways where the chip will be submerged, and the boundary condition will be an unknown temperature that may be just above freezing. Further, the above energy analysis assumes open-loop control with excessive warmup times, whereas the PID controller could reach steady state much faster. The temperature overshoot would need to be minimized to avoid approaching the glass transition temperature of PMMA, as well as to avoid bubble generation in the fluid sample that could affect the optical measurements. PID control was not implemented in this design because it was not necessary for the benchtop proof of concept described in this paper; however, it will be needed when the design is applied to in situ testing. Even when a PID is implemented for in situ deployment, the reaction kinetics for nitrate-to-nitrite reduction will impose a lower limit of several minutes of active heating time. Regardless, the thermal insulation provided by our air pocket design will enable steady state to be attained faster with or without a PID compared to non-insulated designs.

### 3.2. Heater Experimental Characterization

**Figure 3:**
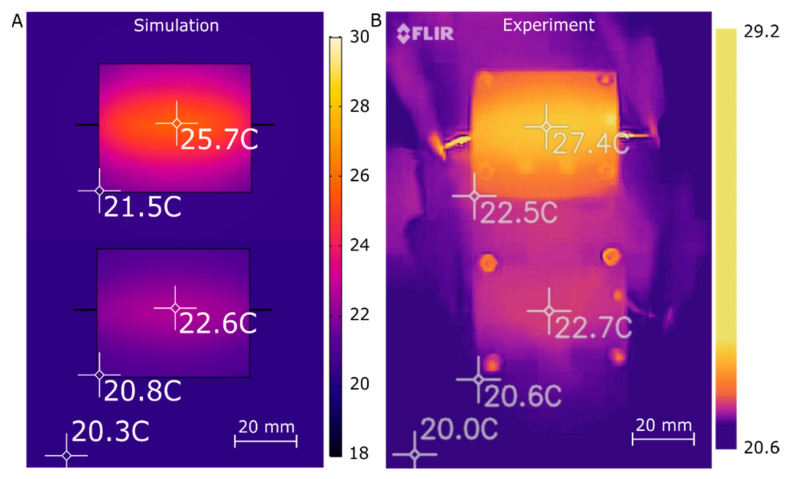
(**A**) Simulated top-down heat distribution of the microfluidic chips. The top rectangle is the standard chip, and the bottom rectangle is the insulated chip; both channel temperatures were set to 35 °C. The crosshairs represent the chip surface temperature at the location in the center of the crosshair; for example, the center of the standard chip is 25.7 °C, whereas the center of the insulated chip is 22.6 °C. (**B**) Experimental top-down thermal image of the heat distributions for the standard (top) and insulated (bottom) microfluidic chips, confirming simulations within the uncertainty of the measurements [38].

Surface thermal characterization of the microfluidic devices is shown in Figure 3, which depicts a top-down view of the standard and insulated chips, both in the simulation and as measured experimentally. Figure 3A shows the simulated results with the standard chip at the top of the image and the insulated chip at the bottom of the image. Crosshairs were placed at both the center and bottom left-hand corner of both chips. The crosshairs were labeled with the readings at their centers and were placed such that they would record the maximum and minimum temperatures of the chip. To confirm the accuracy of our simulations, the surface temperature was recorded using a FLIR one thermal camera. The acquired thermal image, displayed in Figure 3B, shows the two built designs: the standard chip and the insulated chip. As with Figure 3A,B shows the standard chip at the top and the insulated chip on the bottom with the temperatures at the center and bottom left corner marked with crosshairs. In both the simulation and experiment, the nichrome wire heater was given 0.618 A in the standard chip and 0.393 A in the insulated chip. The current values were chosen to bring the fluid channel from 20 °C to 35 °C as predicted by the simulation. The standard chip’s temperature ranges from 21.5 °C to 25.7 °C in the simulation and 22.5 °C to 27.4 °C in the experiment. The experimental temperatures for the standard chip are higher than the simulations by 1.0 °C at the lower left corner and 1.7 °C at the center. The surface temperature of the insulated chip ranged from 20.8 °C to 22.6 °C in the simulation and 20.6 °C to 22.7 °C in the experiment. The insulated chip showed excellent agreement with the simulations, with a 0.2 °C difference at the corner and 0.1 °C at the center. The differences noted are within the ±2 °C accuracy of the FLIR one camera, indicating that simulations and experimental results had comparable surface heat distributions [38].

The surface of the insulated chip is cooler than the surface of the standard chip by 3.1 °C in the simulation and 4.7 °C in the experiment at the center of each chip. This result is expected, since the insulated chip requires less power to maintain a temperature of 35 °C in its fluid channel in steady state conditions. Therefore, less heat is dissipated on the surface. These modeling efforts will enable future optimization as they reflect the experimental observations very well.

**Figure 4:**
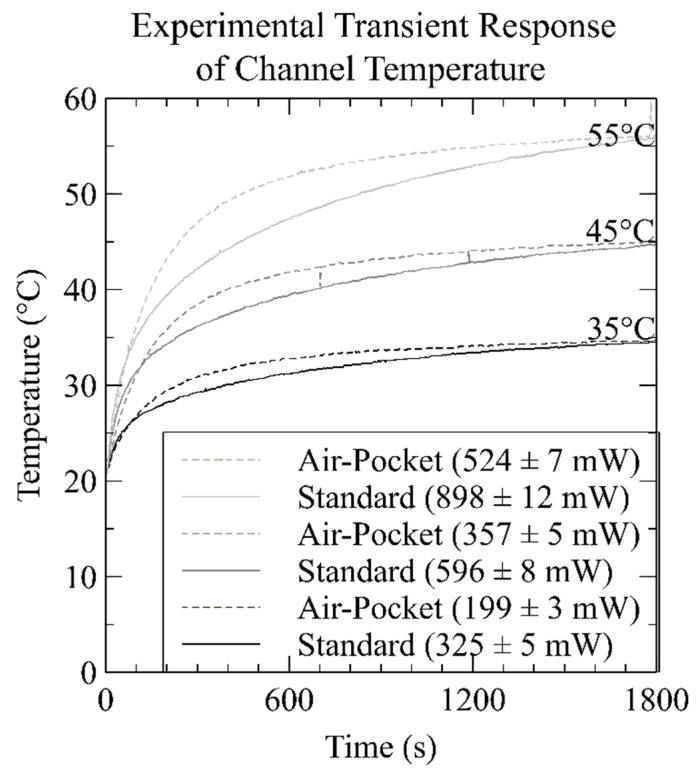
Experimental comparison of the transient responses of the fabricated microfluidic devices. The standard chip (solid lines) and the insulated chip (dashed lines) were evaluated at 3 temperature setpoints of 35 °C, 45 °C, and 55 °C. The air pockets reduced power consumption by 38.9% for the 35 °C temperature setpoint, of 40.0% for the 45 °C, and 41.6% for the 55 °C.

Figure 4 shows the measured transient heat response of both the standard and insulated chips at three temperature setpoints. In the case of the standard chip with a setpoint of 35 °C (black lines), the heater wire in the standard chip was driven at a constant current of 0.65 A and the temperature at the center of its fluid channel was monitored using a k-type thermocouple (TP870, Extech, Boston, MA, USA) for 30 min. In total, 600 temperature measurements were acquired over the duration of the test. The temperature started at 20 °C, or room temperature, and rose to 35 °C, beginning to plateau after 10 min. For the other two temperature setpoints, 0.88 A and 1.08 A were used, and correspondingly, the temperature at the center of the same chip was observed to rise from 20 °C to 45 °C (dark grey lines), and from 20 °C to 55 °C (light grey lines). The same procedure was repeated for the insulated chip, where 0.51 A, 0.68 A, and 0.82 A were used to evaluate the transient response for the same three temperature setpoints.

The devices tested in Figure 4 were different than those of Figure 2 and Figure 3, in that a thermocouple port was added as described in material and methods to monitor the transient response of the channel temperature. The modifications to the chip, and the added thermocouple, changed the thermal properties of the device and the current setpoints were increased to attain the desired steady-state temperatures in the fluid channel. It was empirically determined that to reach the three temperature setpoints of 35 °C, 45 °C, and 55 °C, the standard chip required input powers of 325 ± 5 mW, 596 ± 8 mW, and 898 ± 12 mW, respectively. Similarly, to reach the same temperatures, the insulated chip required 199 ± 3 mW, 357 ± 5 mW, and 524 ± 7 mW, respectively. The error in power was calculated from the combined measurement error values for current and resistance measurements given in the multimeter (5491B, B&K Precision, Yorba Linda, CA, USA) datasheet. The data from these experiments show that we were able to reduce power consumption by 38.9% for the 35 °C temperature setpoint, by 40.0% for the 45 °C setpoint, and by 41.6% for the 55 °C setpoint. 

When comparing the experimental results in Figure 4 to the simulations of Figure 2, the power required to reach 35°C, 45 °C, and 55 °C was 12.1%, 21.7%, and 29.3% higher in the experiment than in simulations for the standard chip. Similarly, the power required was 69.5%, 78.7% and 85.5% higher in the experiment than in simulations for the insulated chip. Therefore, instead of a 60% average reduction in energy as predicted by simulations when implementing the air pocket design, we only observe approximately 40% average reduction in energy in the experiment. We attribute the discrepancy in energy reduction to two differences between the simulation and experiment. First, the initial simulations did not include the added thermal mass of the thermocouple. Second, to bring the thermocouple into communication with the channel, we were required to break the insulating air pockets, thus permitting convection and additional heat dissipation through the thermocouple port. To simulate our experimental setup more accurately, we modified the simulations to include the thermocouple mass. In the supplementary material, Section B, we evaluated the impact of adding the thermocouple to the simulations in Figure 2 and show our results in Figure S2. The correct size and material for the k-type thermocouple were included in the simulation, and ultimately, provided an additional lower resistance path for the heat energy to transfer out of the chips. When the simulation included the thermocouple, the power required to reach 35 °C, 45 °C, and 55 °C were 350 mW (290 mW originally), 596 mW (490 mW originally), and 838 mW (695 mW originally) in the standard chip, and 177 mW (117 mW originally), 301 mW (200 mW originally), and 428 mW (283 mW originally) in the insulated chip, respectively. On average, the addition of the thermocouple in the simulation increased the power required by 20.8% for the standard chip and 50.9% for the insulated chip. This reduced the average discrepancy between simulation and experiment from 21.0 to 0.1% in the standard chip and 77.9 to 17.9% in the insulated chip, where the new simulated powers reflect those shown in Figure 4’s caption. A summary of the above data is provided in Table 1. Therefore, the addition of the thermocouple accounts for the majority of the noted difference. The remaining minor difference is likely due to convection through the hole drilled for thermocouple access to the fluid channel. With our well-characterized heater design, we next proceeded to apply the microfluidic devices to perform colorimetric assays for nitrate.

### 3.3. On-Chip Measurement of Nitrate Using the Inlaid Optical Cell

**Figure 5:**
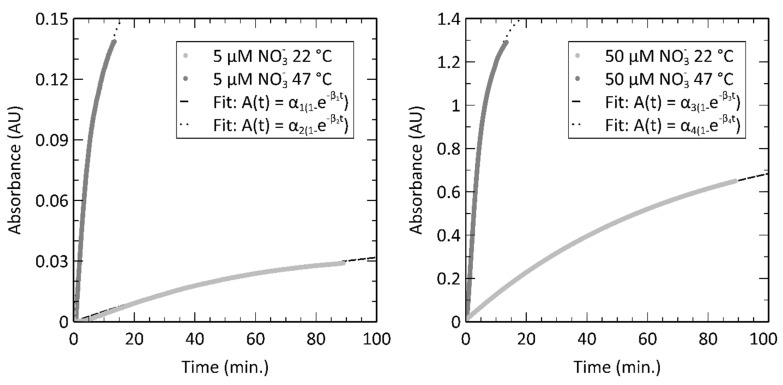
Reaction kinetics studied over 100 min for the vanadium reduction and Griess nitrate color development assay described in the Materials and Methods. Absorbance measured at 527 nm versus time for a 5 µM nitrate concentration at 22 °C (RT) and at 47 °C, highlighting the slow color development at low temperatures. The absorbance was 29 mAU after 90 min at 22 °C, whereas when heated to 47 °C, the same concentration resulted in an absorbance of 125 mAU after only 10 min. Fits to an exponential function are also shown, representing first-order reaction kinetics as described in text. Similar experimental study with a 50 µM nitrate concentration. High concentrations still benefit from heating, but not to the same degree as the lower concentrations.

Before proceeding to perform the vanadium reduction assay with our on-chip heater, we first characterized the assay on-chip using an externally driven temperature source in the form of a custom enclosure around a hotplate. In other words, we established stable temperature setpoints using an off-chip heater but performed the chemistry and optical measurement within the inlaid microfluidic channel. Figure 5 shows the measured absorbance values at 527 nm over time, conducted in a simple inlaid optical absorbance cell in a thermally regulated chamber set at two temperatures. The nitrate colorimetric reaction was characterized by using a low (5 µM) and a high (50 µM) concentration of nitrate mixed in a 1:1 volumetric ratio with the modified Griess reagent at room temperature (22 °C) and heated conditions (47 °C). A setpoint of 47 °C was selected as our maximum temperature because we observed that higher temperatures generated bubbles in the fluid that would interfere with the optical measurements unless sufficient degassing of fluids was performed. The formation of the colored Azo dye is a two-step chemical reaction, where nitrate is first reduced to nitrite by vanadium (III) chloride; then, the nitrite reacts with the Griess reagent to form the Azo dye. The rate kinetics are largely dominated by the vanadium reduction reaction, as the Griess reaction is relatively quick, reaching 95% completion in under a minute at room temperature [22]. Therefore, the duration of color development shown in Figure 5 is rate-limited primarily from the vanadium reduction in the two-step process.

We estimate the time to 95% completion using a single exponential fit, given that the time constant for the Griess reaction is significantly faster than the nitrate-to-nitrite reduction. For the 5 µM sample at 22 °C, α_1_ = 0.0455, β_1_ = 0.0002, and for the 50 µM sample at 22 °C, α_2_ = 0.1737, β_2_ = 0.00211. For the 5 µM sample at 47 °C, α_3_ = 0.8835, β_3_ = 0.000248, and for the 50 µM sample at 47 °C, α_4_ = 1.468, β_4_ = 0.00283. The extrapolation of this fit indicates that it would take 225 min to reach 95% completion at room temperature. Similarly, it would take 20 min to reach 95% completion at 47 °C. The reaction kinetics of the vanadium method for nitrate-to-nitrite reduction has been previously studied by Schnetger et al. at similar temperatures [2]. Schnetger evaluated the reaction kinetics at 23 °C and 45 °C, finding that the time required to reach 95% completion was 358 min and 31 min; or 91.3% less time to achieve the same color development when heated. We observe similar improvements in reaction kinetics as shown by the data in Figure 5 and the extrapolated time for 95% color development, down from 225 min at 22 °C to 20 min at 47 °C, or 91.1% less time. Therefore, to save time and energy, it will be imperative to heat the sample for as little time as possible, whilst ensuring sufficient time for color development.

We arbitrarily selected 10 min of development time for future experiments in this manuscript. While absorbance values are unitless, we have labeled them as AU or absorbance units for the sake of clarity. The data from Figure 5 show that the 22 °C experiments at 10 min resulted in absorbance values of 3.78 mAU for the 5 µM sample and 125 mAU for the 50 µM sample. Therefore, a 10 min development time without heating yielded insufficient color development for most oceanographic or in situ sensing purposes, as our setup typically is characterized by a noise floor of ±1 mAU, or a lower limit of 1–2 µM without heating. However, at 47 °C and 10 min, the absorbances from Figure 5 were 125 mAU for the 5 µM sample and 1.20 AU for the 50 µM sample. The increased color development indicates that a nanomolar limit of detection is possible with heating. 

### 3.4. Nitrate Detection Using Integral Heater with Inlaid Optical Cell

**Figure 6:**
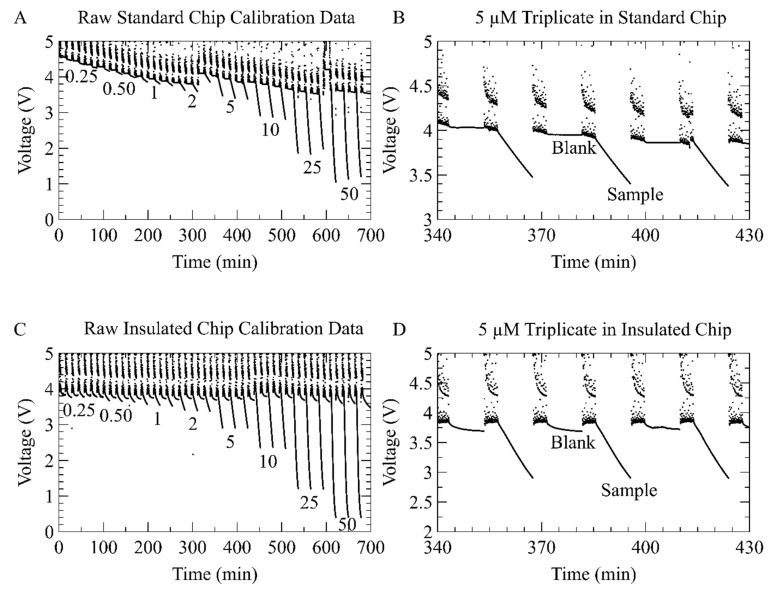
Raw photodiode voltage output over time collected during the calibration procedure. (**A**) The voltage output from the standard chip calibration. Each of the 8 sample concentrations were tested three times with blank measurements taken between each sample. As expected, higher concentrations cause larger drops in voltage. A downward drift is visible in the data and is discussed in the text. (**B**) A zoomed in view of the standard chip raw voltage data showing only the 5 µM triplicate. The second 5 µM sample and its associated blank measurement are labeled. Preceding each blank and each sample is a period of noise caused by fluid moving through the optical channel during the pumping process. (**C**) Voltage output from the insulated chip calibration. The downward drift visible in the standard chip calibration was not present; however, there was some crosstalk between samples. (**D**) Zoomed in view of the insulated chip data for the 5 µM triplicate.

The chips with integrated heaters were then utilized to perform a calibration using eight nitrate standards with concentrations from 0.25 µM to 50 µM. Figure 6 shows the raw voltage data gathered from the photodiodes from both the standard and insulated chips for all eight standards run in triplicate. The procedure for this test is described in brief here and in detail in Section 2.3. Light was directed through a 25 mm-long microfluidic channel and detected by a photodiode on the other end of the channel. The samples form Azo dye in proportion to the concentration of nitrate, thereby reducing the amount of light that reaches the photodiode and decreasing its voltage output. Figure 6A shows the entire data sequence collected on the standard chip, of eight concentrations measured in triplicate, totaling 24 sample measurements and 24 blank measurements. Figure 6B shows a plot of the same data from Figure 6A, but zoomed in on the triplicate of the 5 µM samples. The second 5 µM sample in Figure 6B labels the blank measurement and the sample measurement. The plateaus in Figure 6B represent the blank (10 min per), the decaying voltage represents the sample color development (10 min per), and the stochastic data between plateaus and decays represent the time where pumping (4 min per) is occurring. During the pumping phase, a bi-modal data trend is noted; when the fluid is stopped, the voltage reading is approximately that of a blank and when fluid is being pumped, the voltage is approximately 0.4 V higher due to Schlieren effect arising from a parabolic pressure-driven flow profile. The withdraw and inject sequence of the syringe pumping also introduces a small amount of noise about these two-levels (fluid stopped and fluid pumping). Similarly, Figure 6C shows the entire data sequence collected on the insulated chip, of eight concentrations measured in triplicate, totaling 24 sample measurements and 24 blank measurements. Figure 6D shows a plot of the same data from Figure 6C, but zoomed in on the triplicate of the 5 µM samples. The same trends are noted for pumping, blanks, and samples.

The voltage of the blanks in the standard chip gradually dropped over the course of the 700 min experiment in Figure 6A, from a starting value of 4.6 V down to a final value of 3.5 V. We were not able to determine the origin of the 1.1 V downward drift; however, we believe that a bubble was nucleating in the channel. This is partially supported by the rapid jump in the blanks after the second 2 µM measurement; the blank rises instantaneously from 3.8 V to 4.1 V. Again, a jump in the blank preceding the first 50 µM sample, where signal jumped from 3.5 V to 4.2 V then fell back to 3.6 V for the next blank. Microfluidic devices are prone to small bubbles entering the chip and obscuring optical measurements [40]. However, the downward drift in Figure 6A did not affect the absorbance calculations, as blank measurements were acquired before every sample measurement. The insulated chip had a negligible downward drift in blank values, shown in Figure 6C, starting at 3.8 V and falling to 3.6 V over the 700 min. A small degree of crosstalk between samples is observable in Figure 6C, where the blank measurements show color development toward the higher concentrations.

**Figure 7:**
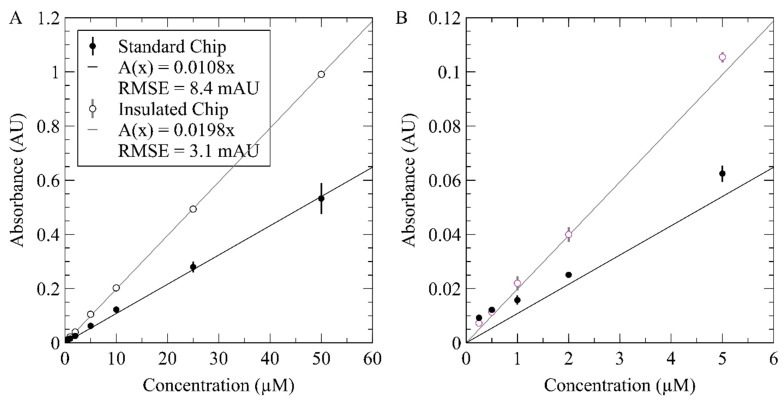
Processed calibration data demonstrating a linear relationship between absorbance and concentration in both the standard and insulated chips. Standard chip was tested at 38 °C while the insulated chip was held at 43 °C. (**A**) Full calibration curve showing concentrations ranging from 0.25 µM to 50 µM. (**B**) Zoomed in calibration curve showing concentrations from 0.25 µM to 5 µM, which can be hard to visualize on the full-scale calibration curve.

To evaluate the expected linear relationship as per the Beer–Lambert law, the raw data from Figure 6A and C were used to calculate the absorbance values versus concentrations. Figure 7 shows the measured absorbance values for each sample plotted against their concentrations. The standard chip data are shown as solid black circles, while the insulated chip data are shown as black outlines around white circles. Figure 7A shows the entire range of concentrations tested and Figure 7B plots the first five concentrations from 0.25 µM to 5 µM. Each data point in Figure 7 is an average of the three consecutive absorbance measurements. Standard deviations are calculated and included for each point; however, they are often not visible, as values are typically below 10 mAU in Figure 7A and below 2 mAU in Figure 7B. The TSL257 photodiode’s datasheet reports a measurement error of 200 µVrms. With the 5V blank voltage here, this would lead to a 10^−5^ AU error, making the optical error negligible compared to the triplicate standard deviation. A linear fit for each dataset is also shown in Figure 7A,B. The line of best fit was found to be *A*(*x*) = 0.0108*x* for the standard chip and *A*(*x*) = 0.0198*x* for the insulated chip, where *x* is the nitrate concentration in µM and *A*(*x*) is the Absorbance value in AU at a given nitrate concentration. Both lines were held to a y-intercept of 0 during fitting in accordance with the Beer–Lambert law outlined in Equation (2). The fits had R-squared values of better than 0.99, and a root mean square error (RMSE) of 8.4 mAU for the standard chip and 3.1 mAU for the insulated chip. These metrics confirm the expected linear relationship.

From the Beer–Lambert law, the slope of the absorbance versus concentration relationship is the product of the length of the optical interrogation channel (25 mm) and the molar attenuation coefficient of the species at a fixed wavelength. The slopes of the standard and insulated chip’s calibration data result in molar attenuation coefficients of 0.00432 (µM cm)^−1^ and 0.00792 (µM cm)^−1^, respectively. The coefficients from our experiments are lower than the reported value of 0.0269 (µM cm)^−1^ by Luy et al. [22]. The main contributor to our underreported molar attenuation coefficients is the incomplete color development. Here, measurements were taken after 10 min and not given time to react fully, whereas in Luy et al., the samples were premixed and left for several hours. The difference between the two attenuation coefficients (i.e., the slopes of Figure 7) is also attributed to varying degrees of color development completion. The reactions reached different levels of completion in the standard and insulated chips. Both chips were driven with the currents that were determined to achieve a channel temperature of 35 °C in Figure 4; however, those current setpoints were determined in chips with integrated thermocouples, while the calibration curves were performed in chips without thermocouples. The thermocouple increased the thermal mass as outlined above and described in Section B of the supplementary material. The removal of the thermocouple in Figure 6 and Figure 7 leads to the insulated chips’ calibration curve being performed at an estimated 43 °C, while the standard chip calibration curve was performed at an estimated 38 °C. The higher temperature in the insulated chip caused the nitrate-to-nitrite reduction to occur more quickly and, as a result, increased the absorbance reading for each concentration. At both temperatures, the linear relationship is preserved, indicating successful acceleration of the reaction kinetics in an on-chip optical cell using an integral heater. 

While this demonstration was useful as a proof-of-concept demonstration, our future work will see the implementation of a closed-loop temperature control system. Optically monitoring thermochromic crystals embedded in the channel is a promising alternative to the thermocouple for closing the loop. Thermochromic crystal solutions change their color based on the fluid temperature and would allow the temperature to be monitored optically, removing the need for physical coupling between the heated and non-heated areas of the microfluidic chip [41]. Other methods of wireless temperature reading are also available. Subthreshold ring oscillators have been demonstrated as a means of generating a signal with a temperature-dependent frequency that could be transmitted wirelessly through the air pocket [42]. A source-measurement-unit (SMU) could also be implemented using the relationship between the wire temperature and its resistivity, as demonstrated for platinum [43]. Using the SMU method, the temperature at the heater can be monitored by reading its resistance and allows for a much simpler temperature sensor than the wireless alternatives without requiring any additional physical connection between the suspended optical cell and the rest of the chip. Of these methods, the SMU approach is the most amendable to integration with our suspended optical heater design, given that the same heating wire can be used as the sensing element.

Finally, we determine the limit-of-detection (LOD) for this novel inlaid microfluidic optical cell with an integral heater. The LOD was determined using the triple-sigma literature method [22,44,45], which uses three times the standard deviation observed during blank measurements. From these standard deviations, the LOD of our system was determined to be 5 nM for the standard chip and 20 nM for the insulated chip. The average standard deviation of each blank was 0.196 mAU for the standard chip and 1.170 mAU for the insulated chip. Ultimately, we have demonstrated our novel microfluidic architecture for performing thermally regulated optical absorbance measurements on-chip that achieves nanomolar LODs and improves energy efficiency by at least 40% using integral air pockets.

## 4. Conclusions

We have demonstrated a method for integrating heaters with inlaid microfluidic optical cells for performing absorbance spectroscopy in isothermal conditions. As a model colorimetric assay, we showed the importance of temperature control for completing nitrate-to-nitrite reduction in a reasonable timeframe by comparing reaction kinetics at room temperature and 47 °C. We showed that by creating integral air pockets within the microfluidic device, we could create a suspended bridge that increased the energy efficiency of heating. By surrounding the reaction chamber with an air pocket, less heat energy can escape the chamber and be lost to the environment. Using this method, we were able to reduce the power draw of our heater by 60% in the simulation. The simulated chips showed a reduction in power required to maintain 35 °C from 290 mW to 117 mW. The standard and insulated chip designs were built and tested, showing an approximately 40% reduction in required power, with the discrepancy accounted for by the thermocouple added to monitor the channel temperature. The experimental chips showed a reduction in power required to maintain 35 °C from 325 mW to 199 mW. The chips, which contain 25 mm long 4 µL volume absorbance cells, were then calibrated for nitrate detection and found to be capable of sensing nanomolar nitrate concentrations. The presented design is broadly applicable to many microfluidic systems that require heating and may be limited in available power, particularly in situ systems that run on battery power for extended periods of time. 

## Figures and Tables

**Table 1:** Power required to reach the three temperature set points in the basic simulation, simulation with thermocouple, and experiment.

Temperature (°C)	Simulation (mW)		Simulation with Thermocouple (mW)		Experiment (mW)	
	Standard	Insulated	Standard	Insulated	Standard	Insulated
35	290	117	350	177	325	199
45	490	200	596	301	596	357
55	695	283	838	428	898	524

## Supplementary Materials

The following are available online at https://www.mdpi.com/article/10.3390/mi12080861/s1, Figure S1: Simulation in Water, Figure S2: Simulation Including Thermocouple, Figure S3: Thermal Equivalent Circuit, Figure S4: Thermal Equivalent Circuit Transient Response, Table S1: Material Properties.
