# Supplementary material for: An Energy Efficient Thermally Regulated Optical Spectroscopy Cell for Lab-on-Chip Devices: Applied to Nitrate Detection
Source: Micromachines (Basel). 2021 Jul 22;12(8):861. doi: 10.3390/mi12080861 (PMC8399308; doi:10.3390/mi12080861)
Supplement: Supplementary file 1 [file micromachines-12-00861-s001.zip › micromachines-1282976-SI.pdf]

An energy efficient thermally regulated optical spectroscopy cell for lab-on-chip devices: applied to nitrate detection

Benjamin J. Murphy[1], Edward A. Luy[1], Katerina L. Panzica[1], Gregory Johnson[2], and Vincent J. Sieben[1*]

[1] Department of Electrical and Computer Engineering, Dalhousie University, 1360 Barrington Street, Halifax, Nova Scotia, Canada B3H 4R2

[2] RBR Limited, 359 Terry Fox Drive, Ottawa, Ontario, Canada, K2K 2E7

* Corresponding author: sieben@dal.ca

Section A: The microfluidic chip was simulated in seawater at 4 °C to better model the chip under oceanic conditions. This section outlines the cross-sectional heat distribution of the chip under these conditions as well as the effect on required energy caused by this change in conditions.

Section B: The addition of a thermocouple to the microfluidic chip design was investigated to account for the discrepancy that was found between our simulated and experimental results.

Section C: Both chips were modeled as thermal equivalent RC circuits to confirm the transient responses found in our COMSOL simulation. The equivalent circuits were found to give similar Transient responses to those found both in simulation and in experiment.

A Simulation at 4 °C and submersed in water

Figure S1 shows the heat distribution of both the standard (A) and insulated (B) chips simulated at 4 °C submerged in seawater. As in the simulations shown in Figure 2 the air pockets caused the heat distribution to change significantly. The temperature in both chips is highest at the heater and decays as it approaches the edges. In the standard chip this decay was gradual throughout the chip while in the insulated chip the temperature drop occurred primarily within the air pocket.

The simulation in 4 °C seawater differs from the simulation in 20 °C air primarily in the amount of power that was required to heat the chip and the temperature that the heater had to reach to achieve the desired temperatures. To achieve the channel temperature setpoints of 35 °C, 45 °C, and 55°C in oceanic conditions the standard chip required 0.727 W, 0.965 W, and 1.20 W while the insulated chip required 0.255 W, 0.340 W, and 0.428 W. This is a significant increase in required power over the simulation of the chip in 20 °C air that required 2.90 W, 0.490 W, and 0.695 W in the standard chip and 0.117 W, 0.200 W, and 0.283 W in the insulated chip. This increase in required power is the expected result of having to maintain a 16 °C higher difference between channel temperature and ambient temperature. Additionally, the thermal conductivity of water (0.6 W/m*K) is much higher than the thermal conductivity of air (0.026 W/m*K) which leads to more energy being lost to convection in water. The efficiency improvement from the added insulation reduced the power required to heat the chip by 65.0 %, 64.~~5~~8 %, and 64.5% under oceanic conditions.

The increase in power required is accompanied by an increase in heater temperature. For our 3 temperature setpoints, the simulation in 4 °C seawater required heater wire temperatures of 50.8 °C, 65.8 °C, and 80.7 °C in the standard chip and 40.7 °C, 52.7 °C, and 64.6 °C in the insulated chip. This is higher than the heater temperatures when the system is simulated in air at 20 °C of 41.7 °C, 56.3 °C, and 70.9 °C in the standard chip and 37.8 °C, 50.1 °C, and 62.0 °C in the insulated chip. While the heater temperatures are higher in the simulations performed in 4 °C seawater the temperatures do not approach the glass transition temperature of PMMA of 100 °C to 130 °C so this chip material would still function under these conditions.

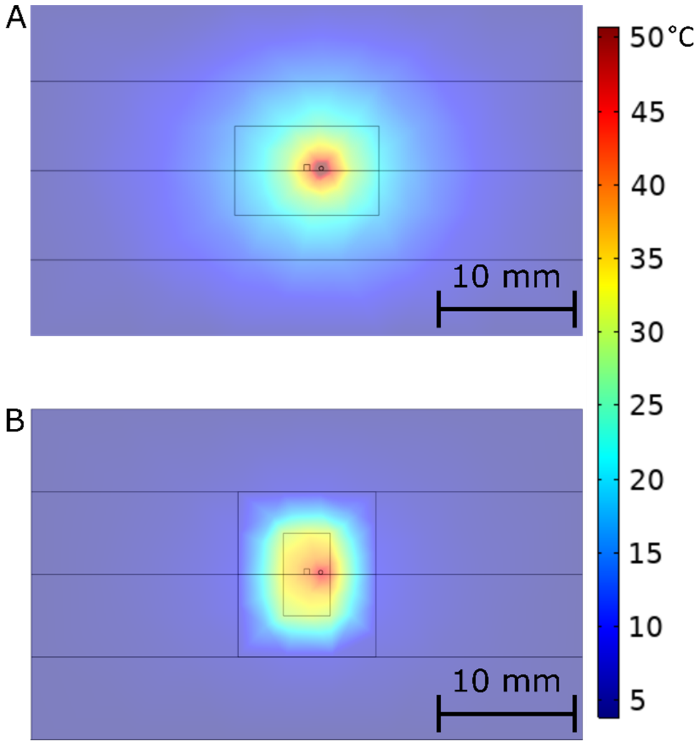

Figure S1: (A) Simulated cross-sectional heat distribution for the standard chip submerged in 4 °C water. The temperature setpoint is 35 °C at the center of the fluid channel and required 0.978 A supplied to the heater, which itself attained a temperature of 50.8 °C. (B) Simulated cross-sectional heat distribution in the chip at steady-state conditions with insulating air pockets surrounding the fluid channel and heater. The same 35 °C channel setpoint required 0.579 A supplied to the heater, with the heater reaching 40.7 °C.

B Simulation including the thermocouple

Figure S2 shows the cross-sectional heat distribution when a thermocouple is added at the center of the chip to read the temperature in the fluid channel. The heat distribution changes significantly with the addition of the thermocouple. The thermocouple provides an easier path for heat to escape through the top of this chip. This is evident in the heat distribution of both chips but is particularly noticeable in the insulated chip where the thermocouple provides a way for the heat to bypass the insulation entirely leading to significantly higher temperatures outside of the suspended bridge and considerably more heat energy lost to the environment.

The k-type thermocouple (TP870, Extech, Boston, USA) was modeled as a 2 mm diameter silicone sheath around a 0.3 mm diameter cylinder of Chromel (90 % nickel, 10 % Chromium) and a 0.3 mm diameter cylinder of Alumel (95 % nickel, 2 % aluminum, 2 % manganese, 1 % silicon). This thermocouple was placed in a hole drilled 12 mm deep at the center of the top surface of each chip.

The addition of the thermocouple increases the power required to reach 35 °C, 45 °C, and 55 °C in both chips. In the standard chip the required power increased from 0.290 W, 0.490 W, and 0.695 W without the thermocouple to 0.350 W, 0.594 W, and 0.838 W with the thermocouple. The thermocouple increased the power required to reach each of the temperature setpoints in the standard chip by 20.7 %, 21.2 %, and 20.6 % respectively. In the insulated chip the required power increased from 0.117 W, 0.200 W, and 0.283 W without the thermocouple to 0.177 W, 0.301 W, and 0.428 W with the thermocouple. The thermocouple increased the required power to reach each of the temperature setpoints in the insulated chip by 51.~~3~~0 %, 50.~~5~~3 %, and 51.2 % respectively. The effect of the thermocouple is more significant in the insulated chip with an average increase in required power of ~~51.0~~50.9 % compared the average increase in required power of 20.8 % in the standard chip. The thermocouple having a larger effect on the insulated design than it does on the standard design causes the energy savings from the added insulation to be only approximately 40 % compared to the approximately 60 % energy savings in the simulations without thermocouples.

The experimental results in Figure 4 were gathered using a chip with an integrated thermocouple similar to the one used in this simulation. The required power values to reach steady state channel temperatures of 35 °C, 45 °C, and 55 °C were experimentally determined to be 0.325 W, 0.596 W, and 0.898 W for the standard chip and 0.199 W, 0.35~~7~~8 W, and 0.524 W for the insulated chip. The experimental values for the standard chip when compared to the values from the simulation with thermocouple are 7.15 % lower at 35 °C, 0.40 % higher at 45 °C, and 7.19 % higher at 55 °C. Similarly, the experimental values for the insulated chip are 12.2 % higher at 35 °C, 18.9 % higher at 45 °C and 22.7 % higher at 55 °C.

The reduction in energy efficiency caused by the addition of the thermocouple makes them a poor method for characterization and for closing the loop on temperature control in heated microfluidic chips. Other methods such as thermochromic crystals and source measurement units would be more effective as they do not require physical coupling and therefore would not affect the heat retention of the chip.

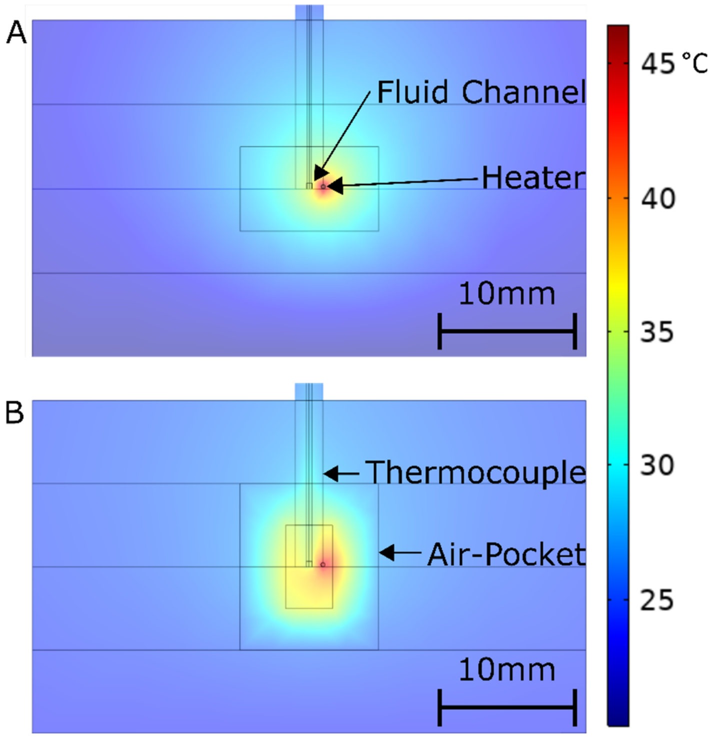

Figure S2: (A) Simulated cross-sectional heat distribution for the standard chip in 20 °C air. The temperature setpoint is 35 °C at the center of the fluid channel. The thermocouple is visible coming down from the top of the chip into the fluid channel. (B) Simulated cross-sectional heat distribution in the chip at steady-state conditions with insulating air pockets surrounding the fluid channel and heater. The thermocouple again comes down from the top of the chip passes through the insulating air-pocket into the fluid channel at the center of the chip.

C Electrical equivalent model

The standard and insulated chips were both modeled as a set of equivalent thermal resistances and capacitances, following the N-stage RC transmission-line equivalent circuit model of the heat-diffusion equation covered by Bruus [1]. This model is an infinite parallel-plate approximation, and as such, under-represents the true 3-dimensional heat transfer problem for our chip geometries. For this reason, we do not expect identical transient and steady-state voltages compared to those reported by COMSOL, but we do expect a similar order of magnitude.

In a thermal equivalent circuit model, the temperature is modeled as voltage or potential difference, and each material layer of the microfluidic chip can be modeled as a thermal resistor and thermal capacitor to capture heat transfer through the device [1]. Equation S1 shows the thermal resistance (R_{th}) which is defined as the height (h) of the layer divided by the layer's surface area (A) and thermal conductivity (k) [1]. Equation S2 shows the thermal capacitance (C_{th}) which is defined as the height (h) of the layer multiplied by its surface area (A), and its material's density (ρ), and specific heat capacity (c_p) [1]. Equation S3 shows the heat lost to *convection* in the air around the chip which is calculated as a resistance (R_{con}) defined as 1 divided by the heat transfer coefficient (K) of air and the area of the surface of the chip (A). The thermal conductivity, density, and specific heat capacity for PMMA, water, and air are listed in Table S1. With these resistance and capacitance values, we model our microfluidic designs as electrical equivalent circuits.

$$R_{th} = \frac{h}{kA} \tag{S1}$$

$$C_{th} = hA\rho c_p \tag{S2}$$

$$R_{Con} = \frac{1}{KA} \tag{S3}$$

Table S1:

	k (W/m*K)	ρ (kg/m^3)	C_p (J/K)	Source
PMMA	0.17	1200	1400	[1]
Water	0.6	1000	4200	[1]
Air	0.026	1.2	1000	[2]

The magnitude of the voltage source, which represents the heater, is set to the difference between the temperature at the heater and the ambient temperature of the environment. In the COMSOL simulations outlined in Figure 2 the temperature at the heater required to reach 35 °C in the microfluidic channel is 41.7 °C in the standard chip and 37.8 °C in the insulated chip. When the voltage, which is analogous to temperature, in the equivalent circuit is set to these values the channel temperature is 38.9°C in the standard chip and 36.3°C in the insulated chip. These values

are close to the 35 °C temperature we would expect and indicate that the equivalent circuit and COMSOL simulations are in reasonable agreement about the expected channel temperatures.

The transient thermal response of the microfluidic design was also simulated using circuit equivalence methods. This transient response is compared to the transient response from the simulated chip in Figure S4. In the equivalent circuit, the time it takes for the temperature in the microfluidic channel to reach 95 % of its final temperature is 38 minutes for the standard chip and 5 minutes for the insulated chip. The transient response of the chips in the COMSOL simulations is 33 minutes for the standard chip and 17 minutes for the insulated chip. While the heating time of the standard chip is very similar between simulation and circuit equivalent, the insulated chip was much less so. This is likely due to incorrect modelling of the air pocket as a conductive element, not appropriately capturing the convection that is occurring within the air pocket. Despite the difference in heating time, the circuit equivalent shows the same shape as the finite element method of COMSOL for the transient response. The insulated chip heats much faster than the standard chip and levels off quickly while the standard chip heats more gradually coming to a slower plateau. From these similarities and the similar temperature steady states we concluded that our simulated results were reasonable and the chip could be constructed physically to gather experimental data.

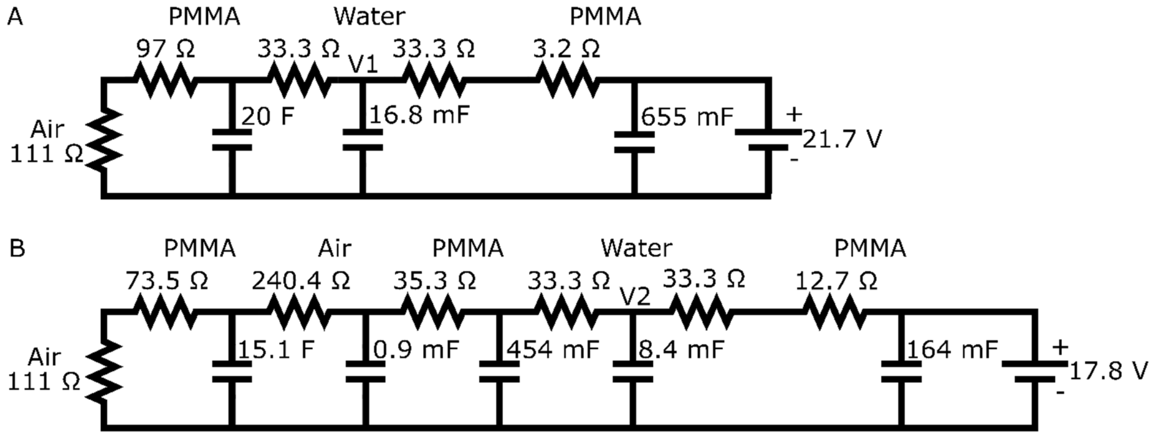

Figure S3: (A) Thermal equivalent circuit of the standard chip design. The heat at the wire passes through only PMMA to reach the water in the microfluidic channel then more PMMA to reach the outside boundary conditions of the chip. The point at the center of the channel where the voltage is measured is marked with V1. (B) Thermal equivalent circuit of the insulated chip design. This circuit is similar to the standard chip circuit with the PMMA broken up by the addition of a thermally resistant air pocket. The point at the center of the channel where the voltage is measured is marked with V2. In both chips ground is defined as room temperature (20 °C), for this reason the potential difference across the voltage sources are the wire temperature minus 20 V.

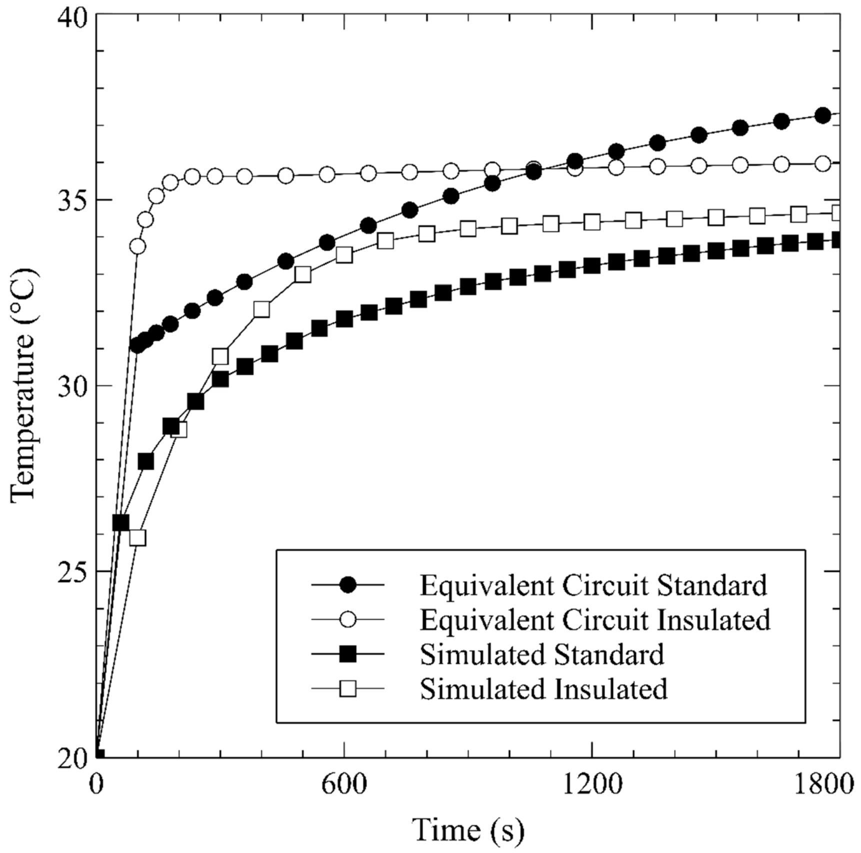

Figure S4: The transient temperature response of the standard chip (solid-filled data points) and insulated chip (non-filled data points) are compared over the first 30 minutes rising from room temperature (20 °C). Additionally, the transient responses simulated in COMSOL (squares) are compared to the transient responses from the circuit equivalent model (circles). Similar transient responses are observed between the COMSOL simulations and the circuit equivalent model. The standard chip gradually approaches its steady state temperature and the insulated chip heats up faster then levels off more sharply towards its steady state temperature.

Supplementary References:

[1] H. Bruus, *Theoretical Microfluidics*, First. Oxford: Oxford University Press, 2008.

[2] Y. Touloukian, R. Powel, C. Ho, and P. Klemens, *Thermophysical Properties of Matter*, vol. Volume 3. Defense Technical Information Center, 1970.
